# Tris(tetra­hydro­furan-κ*O*)tris­[tris­(thio­phen-2-yl)methano­lato-κ*O*]terbium(III) tetra­hydro­furan monosolvate

**DOI:** 10.1107/S160053681104623X

**Published:** 2011-11-09

**Authors:** Michael Veith, Celine Belot, Volker Huch

**Affiliations:** a Institut für Anorganische Chemie, Universität des Saarlandes, Postfach 151150, 66041 Saarbrücken, Germany, and Leibniz Institute for New Materials, 66123 Saarbrücken, Germany

## Abstract

In the mononuclear title compound, [Tb(C_13_H_9_OS_3_)_3_(C_4_H_8_O)_3_]·C_4_H_8_O, the lanthanide cation is located on a threefold rotation axis and is surrounded by electron-rich ligands in an approximately octa­hedral geometry. One of the thienyl groups and the bound THF are disordered with 0.5:0.5 occupancy. The free THF is disordered around the threefold axis.

## Related literature

For the preparation of some other lanthanide alkoxides containing thienyl substituents, see: Veith *et al.* (2008 ▶); Veith, Belot, Huch, Cui *et al.* (2010 ▶). For lanthanide alkoxides, see: Barnhart *et al.* (1993) ▶; Evans *et al.* (1997 ▶, 1999 ▶). For the electrochemical and luminescence properties of 4*f* complexes containing thienyl substituents, see: Teotonio *et al.* (2004 ▶); Viswanathan & de Bettencourt-Dias (2006 ▶); Sultan *et al.* (2006 ▶); Veith, Belot, Huch, Guyard *et al.* (2010 ▶). 
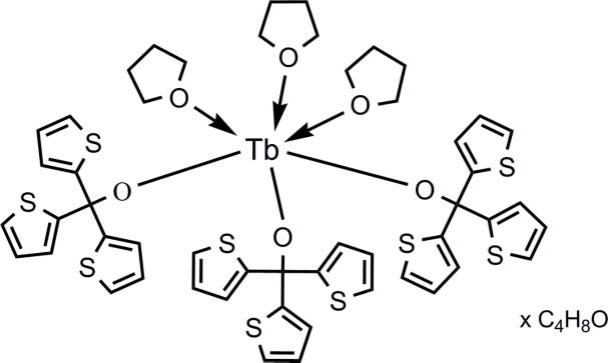


## Experimental

### 

#### Crystal data

[Tb(C_13_H_9_OS_3_)_3_(C_4_H_8_O)_3_]·C_4_H_8_O
*M* *_r_* = 1279.48
Trigonal, 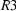

*a* = 13.9131 (4) Å
*c* = 24.6789 (7) Å
*V* = 4137.2 (2) Å^3^
*Z* = 3
Mo *K*α radiation
μ = 1.67 mm^−1^
*T* = 132 K
0.35 × 0.27 × 0.24 mm

#### Data collection

Bruker APEXII CCD diffractometer
Absorption correction: multi-scan (*SADABS*; Bruker, 2010 ▶) *T*_min_ = 0.593, *T*_max_ = 0.693
28927 measured reflections
6212 independent reflections
6197 reflections with *I* > 2σ(*I*)
*R*_int_ = 0.092

#### Refinement

*R*[*F* ^2^ > 2σ(*F* ^2^)] = 0.035
*wR*(*F* ^2^) = 0.085
*S* = 1.04
6212 reflections
260 parameters
16 restraints
H-atom parameters constrained
Δρ_max_ = 1.03 e Å^−3^
Δρ_min_ = −0.63 e Å^−3^
Absolute structure: Flack (1983 ▶), 3011 Friedel pairs
Flack parameter: 0.089 (8)

### 

Data collection: *APEX2* (Bruker, 2010 ▶); cell refinement: *SAINT* (Bruker, 2010 ▶); data reduction: *SAINT*; program(s) used to solve structure: *SHELXS97* (Sheldrick, 2008 ▶); program(s) used to refine structure: *SHELXL97* (Sheldrick, 2008 ▶); molecular graphics: *DIAMOND* (Brandenburg, 1999 ▶); software used to prepare material for publication: *SHELXTL* (Sheldrick, 2008 ▶).

## Supplementary Material

Crystal structure: contains datablock(s) I, global. DOI: 10.1107/S160053681104623X/nc2251sup1.cif

Supplementary material file. DOI: 10.1107/S160053681104623X/nc2251Isup2.cdx

Structure factors: contains datablock(s) I. DOI: 10.1107/S160053681104623X/nc2251Isup3.hkl

Additional supplementary materials:  crystallographic information; 3D view; checkCIF report

## Figures and Tables

**Fig. 1.**
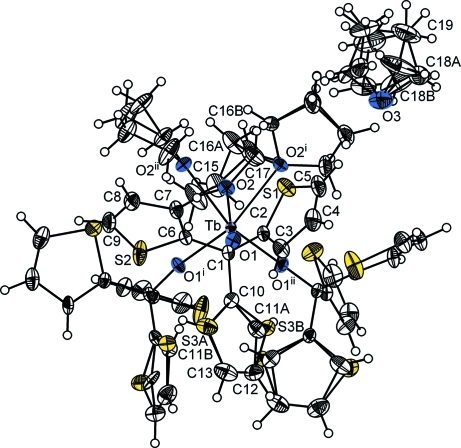
The molecular structure of the title compound with atom labels and 50% probability displacement ellipsoids for non-H atoms. Symmetry codes: i = 1 - y,x-y,zii = 1 - x + y,1 - x,z

**Fig. 2.**
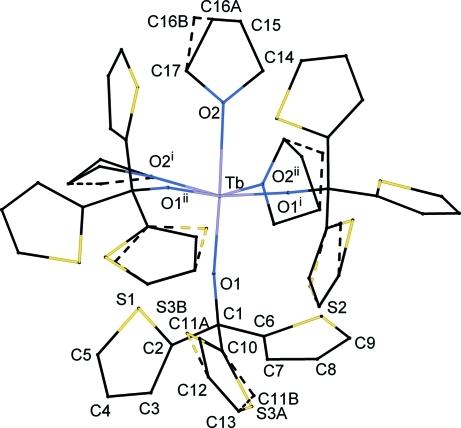
Wire frame model of the title compound. H atoms the free thf molecule have been omitted for clarity and split positions are indicated by dashed lines. Symmetry codes: i = 1- y,x-y,zii = 1 - x + y,1 - x,z

## supplementary crystallographic information

### Comment

The title compound was obtained at the work in synthesizing lanthanide complexes containing thiophene derivatives. The asymmetric unit consists of one third of the complex which is located on a threefold axis (2/3,1/3,*z*) and one third of an additional thf molecule, which is disordered around the threefold axis. The molecular structure reveals a mononuclear compound with an approximately octahedral geometry around the metal centre (Fig. 1 and 2). It is surrounded by three tris(2-thienyl)methoxido ligands and by three tetrahydrofurane molecules in a facial arrangement. This facial coordination geometry around the metal is similar to those described in our previous paper (Veith *et al.*, 2008;  Veith, Belot, Huch, Cui *et al.*, 2010; Veith, Belot, Huch, Guyard *et al.*, 2010). The aditional THF molecule also present in the crystal lattice has no interaction with the molecule.

### Experimental

The title compound was obtained by the reaction between one equivalent (1.12 mmol, 0.719 g) of Tb[N(SiMe_3_)_2_]_3_ in 25 ml thf and three equivalents (3.37 mmol, 0.939 g) of the carbinol tris(2-thienyl)methanol in 25 ml tetrahydrofuran as solvent. The mixture was stirred at room temperature two days, concentrated and placed at 5°C. Few days later crystals were grown.

### Refinement

One of the thienyl rings shows a 180° rotational disorder. S(3) and C(11) were refined with split atom positions. Restraints were used to fulfil the thienyl ring geometry of the disordered positions in the refinement. The free thf-molecule is disordered on the threefold axis. The crystal is racemically twinned and therefore, a twin refinement was performed (BASF parameter: 0.08931). H atoms were positioned geometrically and refined with C—H = 0.95–0.99 Å and with *U*_iso_(H) = 1.2 times *U*_eq_(C).

### Crystal data

**Table:**

[Tb(C_13_H_9_OS_3_)_3_(C_4_H_8_O)_3_]·C_4_H_8_O	*D*_x_ = 1.541 Mg m^−^^3^
*M**_r_* = 1279.48	Mo *K*α radiation, λ = 0.71073 Å
Trigonal, *R*3	Cell parameters from 9799 reflections
*a* = 13.9131 (4) Å	θ = 2.9–32.0°
*c* = 24.6789 (7) Å	µ = 1.67 mm^−^^1^
*V* = 4137.2 (2) Å^3^	*T* = 132 K
*Z* = 3	Block, colourless
*F*(000) = 1962	0.35 × 0.27 × 0.24 mm

### Data collection

**Table:**

Bruker APEXII CCD diffractometer	6212 independent reflections
Radiation source: fine-focus sealed tube	6197 reflections with *I* > 2σ(*I*)
graphite	*R*_int_ = 0.092
φ and ω scans	θ_max_ = 32.0°, θ_min_ = 1.9°
Absorption correction: multi-scan (*SADABS*; Bruker, 2010)	*h* = −20→20
*T*_min_ = 0.593, *T*_max_ = 0.693	*k* = −20→20
28927 measured reflections	*l* = −36→34

### Refinement

**Table:**

Refinement on *F*^2^	Secondary atom site location: difference Fourier map
Least-squares matrix: full	Hydrogen site location: inferred from neighbouring sites
*R*[*F*^2^ > 2σ(*F*^2^)] = 0.035	H-atom parameters constrained
*wR*(*F*^2^) = 0.085	*w* = 1/[σ^2^(*F*_o_^2^) + (0.0501*P*)^2^] where *P* = (*F*_o_^2^ + 2*F*_c_^2^)/3
*S* = 1.04	(Δ/σ)_max_ = 0.001
6212 reflections	Δρ_max_ = 1.03 e Å^−^^3^
260 parameters	Δρ_min_ = −0.63 e Å^−^^3^
16 restraints	Absolute structure: Flack (1983), 3011 Friedel pairs
Primary atom site location: structure-invariant direct methods	Flack parameter: 0.089 (8)

### Special details

**Table:**

Geometry. All e.s.d.'s (except the e.s.d. in the dihedral angle between two l.s. planes) are estimated using the full covariance matrix. The cell e.s.d.'s are taken into account individually in the estimation of e.s.d.'s in distances, angles and torsion angles; correlations between e.s.d.'s in cell parameters are only used when they are defined by crystal symmetry. An approximate (isotropic) treatment of cell e.s.d.'s is used for estimating e.s.d.'s involving l.s. planes.
Refinement. Refinement of *F*^2^ against ALL reflections. The weighted *R*-factor *wR* and goodness of fit *S* are based on *F*^2^, conventional *R*-factors *R* are based on *F*, with *F* set to zero for negative *F*^2^. The threshold expression of *F*^2^ > σ(*F*^2^) is used only for calculating *R*-factors(gt) *etc*. and is not relevant to the choice of reflections for refinement. *R*-factors based on *F*^2^ are statistically about twice as large as those based on *F*, and *R*- factors based on ALL data will be even larger.

### Fractional atomic coordinates and isotropic or equivalent isotropic displacement parameters (Å^2^)

**Table:**

	*x*	*y*	*z*	*U*_iso_*/*U*_eq_	Occ. (<1)
Tb	0.6667	0.3333	0.0413	0.01172 (5)	
O1	0.79633 (18)	0.47581 (17)	0.08025 (10)	0.0197 (4)	
C1	0.8664 (2)	0.5704 (2)	0.10997 (12)	0.0167 (4)	
C2	0.9868 (2)	0.6079 (2)	0.09631 (12)	0.0190 (5)	
C3	1.0824 (2)	0.6674 (3)	0.12849 (16)	0.0288 (7)	
H3	1.0822	0.6906	0.1647	0.035*	
C4	1.1800 (3)	0.6882 (3)	0.0992 (2)	0.0363 (8)	
H4	1.2527	0.7275	0.1140	0.044*	
C5	1.1588 (3)	0.6466 (4)	0.0488 (2)	0.0375 (9)	
H5	1.2143	0.6538	0.0241	0.045*	
S1	1.02039 (8)	0.57944 (10)	0.03432 (4)	0.0351 (2)	
C6	0.8418 (2)	0.6629 (2)	0.09634 (12)	0.0187 (5)	
C7	0.9090 (3)	0.7650 (2)	0.07317 (15)	0.0253 (6)	
H7	0.9852	0.7933	0.0649	0.030*	
C8	0.8506 (4)	0.8236 (3)	0.0629 (2)	0.0325 (8)	
H8	0.8838	0.8946	0.0463	0.039*	
C9	0.7454 (4)	0.7689 (3)	0.07891 (19)	0.0356 (8)	
H9	0.6957	0.7967	0.0757	0.043*	
S2	0.71026 (8)	0.64206 (9)	0.10600 (6)	0.0457 (3)	
C10	0.8529 (3)	0.5479 (3)	0.17053 (15)	0.0199 (5)	
C11A	0.8283 (16)	0.4490 (13)	0.1987 (6)	0.030 (3)	0.56
H11A	0.8100	0.3808	0.1816	0.036*	0.56
C12	0.8348 (4)	0.4655 (4)	0.25796 (17)	0.0389 (9)	
H12	0.8268	0.4123	0.2844	0.047*	
C13	0.8540 (4)	0.5678 (4)	0.26810 (18)	0.0424 (10)	
H13	0.8523	0.5917	0.3040	0.051*	
S3A	0.8801 (5)	0.6495 (4)	0.21652 (15)	0.0368 (10)	0.56
C11B	0.8561 (19)	0.6242 (19)	0.2149 (5)	0.032 (4)	0.44
H11B	0.8589	0.6934	0.2105	0.038*	0.44
S3B	0.8378 (5)	0.4296 (4)	0.19555 (18)	0.0260 (7)	0.44
O2	0.54323 (18)	0.20269 (19)	−0.03269 (10)	0.0211 (4)	
C14	0.4229 (3)	0.1518 (4)	−0.0337 (2)	0.0463 (12)	
H14A	0.4009	0.1996	−0.0535	0.056*	
H14B	0.3933	0.1409	0.0037	0.056*	
C15	0.3793 (4)	0.0435 (4)	−0.0614 (2)	0.0401 (9)	
H15A	0.3016	0.0124	−0.0736	0.048*	
H15B	0.3870	−0.0118	−0.0394	0.048*	
C16A	0.4628 (7)	0.0895 (11)	−0.1084 (4)	0.041 (3)	0.50
H16A	0.4482	0.1378	−0.1324	0.049*	0.50
H16B	0.4616	0.0291	−0.1301	0.049*	0.50
C16B	0.4724 (8)	0.0512 (9)	−0.0970 (6)	0.045 (3)	0.50
H16C	0.4575	0.0568	−0.1358	0.054*	0.50
H16D	0.4818	−0.0141	−0.0918	0.054*	0.50
C17	0.5743 (3)	0.1570 (3)	−0.07751 (15)	0.0311 (7)	
H17A	0.6026	0.1083	−0.0646	0.037*	
H17B	0.6313	0.2164	−0.1006	0.037*	
O3	1.3333	0.6667	−0.0517 (2)	0.0472 (13)	
C18A	1.3679 (16)	0.7658 (11)	−0.0835 (5)	0.044 (3)	0.33
H18A	1.4446	0.8179	−0.0763	0.053*	0.33
H18B	1.3247	0.7999	−0.0742	0.053*	0.33
C18B	1.281 (2)	0.704 (3)	−0.0866 (7)	0.075 (8)	0.33
H18C	1.2850	0.7696	−0.0715	0.090*	0.33
H18D	1.2045	0.6491	−0.0927	0.090*	0.33
C19	1.3533 (12)	0.7336 (10)	−0.1395 (4)	0.074 (3)	0.67
H19A	1.4201	0.7409	−0.1546	0.089*	0.33
H19B	1.3321	0.7787	−0.1599	0.089*	0.33
H19C	1.4188	0.8055	−0.1379	0.089*	0.33
H19D	1.3108	0.7296	−0.1709	0.089*	0.33

### Atomic displacement parameters (Å^2^)

**Table:**

	*U*^11^	*U*^22^	*U*^33^	*U*^12^	*U*^13^	*U*^23^
Tb	0.01092 (5)	0.01092 (5)	0.01332 (7)	0.00546 (3)	0.000	0.000
O1	0.0189 (9)	0.0130 (8)	0.0235 (11)	0.0052 (7)	−0.0053 (8)	−0.0044 (7)
C1	0.0138 (10)	0.0137 (10)	0.0216 (12)	0.0061 (9)	−0.0011 (9)	−0.0025 (9)
C2	0.0156 (11)	0.0169 (11)	0.0252 (13)	0.0086 (9)	0.0015 (9)	0.0004 (9)
C3	0.0110 (11)	0.0334 (16)	0.0367 (17)	0.0071 (11)	−0.0005 (11)	−0.0034 (13)
C4	0.0178 (14)	0.0357 (18)	0.053 (2)	0.0119 (13)	−0.0017 (14)	−0.0001 (17)
C5	0.0255 (16)	0.040 (2)	0.053 (3)	0.0207 (16)	0.0101 (18)	0.006 (2)
S1	0.0271 (4)	0.0457 (5)	0.0319 (4)	0.0179 (4)	0.0020 (3)	−0.0092 (4)
C6	0.0164 (11)	0.0191 (11)	0.0215 (12)	0.0095 (10)	0.0008 (9)	−0.0019 (9)
C7	0.0245 (13)	0.0151 (11)	0.0383 (17)	0.0115 (11)	0.0079 (12)	0.0043 (11)
C8	0.040 (2)	0.0223 (15)	0.041 (2)	0.0198 (15)	0.0095 (17)	0.0079 (15)
C9	0.0390 (19)	0.0344 (18)	0.046 (2)	0.0281 (17)	0.0047 (16)	0.0049 (16)
S2	0.0235 (4)	0.0370 (5)	0.0825 (9)	0.0196 (4)	0.0158 (5)	0.0256 (5)
C10	0.0178 (12)	0.0154 (12)	0.0230 (14)	0.0056 (10)	−0.0037 (11)	−0.0016 (10)
C11A	0.024 (4)	0.032 (7)	0.030 (4)	0.011 (4)	0.002 (3)	−0.005 (4)
C12	0.037 (2)	0.049 (2)	0.0327 (18)	0.0232 (18)	0.0070 (15)	0.0148 (17)
C13	0.041 (2)	0.048 (2)	0.0301 (18)	0.0158 (19)	0.0044 (16)	−0.0073 (17)
S3A	0.044 (3)	0.0330 (17)	0.0282 (12)	0.0154 (16)	0.0046 (10)	−0.0027 (9)
C11B	0.026 (9)	0.045 (12)	0.028 (7)	0.020 (9)	0.019 (5)	0.016 (6)
S3B	0.0308 (16)	0.0257 (14)	0.0248 (12)	0.0165 (10)	0.0004 (9)	0.0016 (10)
O2	0.0186 (9)	0.0222 (10)	0.0196 (11)	0.0079 (8)	−0.0026 (7)	−0.0064 (8)
C14	0.0178 (14)	0.040 (2)	0.065 (3)	0.0025 (14)	0.0038 (16)	−0.029 (2)
C15	0.0283 (17)	0.0326 (18)	0.051 (2)	0.0087 (15)	−0.0061 (16)	−0.0159 (17)
C16A	0.021 (4)	0.049 (6)	0.042 (6)	0.010 (4)	−0.006 (3)	−0.028 (5)
C16B	0.026 (4)	0.039 (5)	0.062 (8)	0.009 (4)	−0.002 (4)	−0.028 (5)
C17	0.0254 (15)	0.0307 (16)	0.0292 (16)	0.0080 (13)	0.0025 (12)	−0.0137 (13)
O3	0.057 (2)	0.057 (2)	0.028 (2)	0.0283 (11)	0.000	0.000
C18A	0.064 (10)	0.046 (7)	0.036 (6)	0.038 (8)	−0.004 (6)	−0.003 (5)
C18B	0.104 (18)	0.15 (2)	0.039 (8)	0.11 (2)	0.021 (10)	0.035 (12)
C19	0.104 (9)	0.090 (8)	0.034 (4)	0.054 (8)	0.007 (6)	0.026 (5)

### Geometric parameters (Å, °)

**Table:**

Tb—O1^i^	2.129 (2)	O2—C14	1.456 (4)
Tb—O1	2.129 (2)	C14—C15	1.481 (6)
Tb—O1^ii^	2.129 (2)	C14—H14A	0.9900
Tb—O2^i^	2.543 (2)	C14—H14B	0.9900
Tb—O2^ii^	2.543 (2)	C15—C16B	1.522 (11)
Tb—O2	2.543 (2)	C15—C16A	1.536 (11)
O1—C1	1.392 (3)	C15—H15A	0.9900
C1—C10	1.519 (5)	C15—H15B	0.9900
C1—C2	1.522 (4)	C16A—C17	1.553 (10)
C1—C6	1.526 (4)	C16A—H16A	0.9900
C2—C3	1.409 (4)	C16A—H16B	0.9900
C2—S1	1.703 (3)	C16B—C17	1.524 (10)
C3—C4	1.435 (5)	C16B—H16C	0.9900
C3—H3	0.9500	C16B—H16D	0.9900
C4—C5	1.340 (7)	C17—H17A	0.9900
C4—H4	0.9500	C17—H17B	0.9900
C5—S1	1.706 (4)	O3—C18B^iii^	1.387 (12)
C5—H5	0.9500	O3—C18B^iv^	1.387 (12)
C6—C7	1.375 (4)	O3—C18B	1.387 (12)
C6—S2	1.720 (3)	O3—C18A^iii^	1.444 (13)
C7—C8	1.432 (5)	O3—C18A^iv^	1.444 (13)
C7—H7	0.9500	O3—C18A	1.444 (13)
C8—C9	1.327 (6)	C18A—C19	1.435 (17)
C8—H8	0.9500	C18A—H18A	0.9599
C9—S2	1.713 (4)	C18A—H18B	0.9599
C9—H9	0.9500	C18A—H18C	1.2167
C10—C11A	1.423 (13)	C18B—C19	1.57 (2)
C10—C11B	1.510 (19)	C18B—C19^iii^	1.70 (3)
C10—S3B	1.670 (5)	C18B—C18B^iv^	1.89 (2)
C10—S3A	1.701 (6)	C18B—C18B^iii^	1.89 (2)
C11A—C12	1.475 (14)	C18B—H18B	1.1962
C11A—H11A	0.9500	C18B—H18C	0.9600
C12—C13	1.334 (6)	C18B—H18D	0.9600
C12—S3B	1.626 (6)	C19—C19^iii^	1.434 (19)
C12—H12	0.9500	C19—C19^iv^	1.435 (19)
C13—C11B	1.523 (19)	C19—C18B^iv^	1.70 (3)
C13—S3A	1.622 (7)	C19—H19A	0.9600
C13—H13	0.9500	C19—H19B	0.9599
C11B—H11B	0.9500	C19—H19C	0.9600
O2—C17	1.445 (4)	C19—H19D	0.9598
			
O1^i^—Tb—O1	101.20 (8)	C17—C16B—H16D	111.1
O1^i^—Tb—O1^ii^	101.20 (8)	H16C—C16B—H16D	109.0
O1—Tb—O1^ii^	101.20 (8)	O2—C17—C16B	109.2 (5)
O1^i^—Tb—O2^i^	160.88 (8)	O2—C17—C16A	102.3 (5)
O1—Tb—O2^i^	91.79 (8)	C16B—C17—C16A	25.2 (6)
O1^ii^—Tb—O2^i^	89.80 (8)	O2—C17—H17A	111.3
O1^i^—Tb—O2^ii^	91.79 (8)	C16B—C17—H17A	86.4
O1—Tb—O2^ii^	89.80 (8)	C16A—C17—H17A	111.3
O1^ii^—Tb—O2^ii^	160.88 (8)	O2—C17—H17B	111.3
O2^i^—Tb—O2^ii^	74.13 (8)	C16B—C17—H17B	126.5
O1^i^—Tb—O2	89.80 (8)	C16A—C17—H17B	111.3
O1—Tb—O2	160.89 (8)	H17A—C17—H17B	109.2
O1^ii^—Tb—O2	91.79 (8)	C18B^iii^—O3—C18B^iv^	85.6 (12)
O2^i^—Tb—O2	74.13 (8)	C18B^iii^—O3—C18B	85.6 (12)
O2^ii^—Tb—O2	74.13 (8)	C18B^iv^—O3—C18B	85.6 (12)
C1—O1—Tb	170.14 (19)	C18B^iii^—O3—C18A^iii^	44.9 (12)
O1—C1—C10	111.6 (2)	C18B^iv^—O3—C18A^iii^	108.7 (10)
O1—C1—C2	109.8 (2)	C18B—O3—C18A^iii^	51.6 (13)
C10—C1—C2	106.9 (2)	C18B^iii^—O3—C18A^iv^	51.6 (13)
O1—C1—C6	109.5 (2)	C18B^iv^—O3—C18A^iv^	44.9 (12)
C10—C1—C6	109.5 (2)	C18B—O3—C18A^iv^	108.7 (10)
C2—C1—C6	109.6 (2)	C18A^iii^—O3—C18A^iv^	93.4 (8)
C3—C2—C1	129.1 (3)	C18B^iii^—O3—C18A	108.7 (10)
C3—C2—S1	110.8 (2)	C18B^iv^—O3—C18A	51.6 (13)
C1—C2—S1	120.1 (2)	C18B—O3—C18A	44.9 (12)
C2—C3—C4	110.7 (3)	C18A^iii^—O3—C18A	93.4 (8)
C2—C3—H3	124.6	C18A^iv^—O3—C18A	93.4 (8)
C4—C3—H3	124.6	C19—C18A—O3	107.2 (10)
C5—C4—C3	113.5 (3)	C19—C18A—H18A	110.0
C5—C4—H4	123.3	O3—C18A—H18A	109.6
C3—C4—H4	123.3	C19—C18A—H18B	110.9
C4—C5—S1	112.1 (3)	O3—C18A—H18B	110.6
C4—C5—H5	124.0	H18A—C18A—H18B	108.5
S1—C5—H5	124.0	C19—C18A—H18C	104.9
C2—S1—C5	92.9 (2)	O3—C18A—H18C	92.3
C7—C6—C1	129.7 (3)	H18A—C18A—H18C	130.0
C7—C6—S2	110.4 (2)	H18B—C18A—H18C	23.2
C1—C6—S2	119.7 (2)	C19—C18A—H19C	38.3
C6—C7—C8	112.0 (3)	O3—C18A—H19C	135.4
C6—C7—H7	124.0	H18A—C18A—H19C	75.0
C8—C7—H7	124.0	H18B—C18A—H19C	109.3
C9—C8—C7	113.4 (3)	H18C—C18A—H19C	119.2
C9—C8—H8	123.3	O3—C18B—C19	102.9 (11)
C7—C8—H8	123.3	O3—C18B—C19^iii^	96.9 (12)
C8—C9—S2	111.8 (3)	C19—C18B—C19^iii^	51.9 (10)
C8—C9—H9	124.1	O3—C18B—C18B^iv^	47.2 (6)
S2—C9—H9	124.1	C19—C18B—C18B^iv^	58.0 (10)
C9—S2—C6	92.31 (17)	C19^iii^—C18B—C18B^iv^	80.3 (7)
C11A—C10—C11B	103.5 (10)	O3—C18B—C18B^iii^	47.2 (6)
C11A—C10—C1	128.7 (6)	C19—C18B—C18B^iii^	83.5 (6)
C11B—C10—C1	127.7 (9)	C19^iii^—C18B—C18B^iii^	51.7 (8)
C11A—C10—S3B	9.9 (7)	C18B^iv^—C18B—C18B^iii^	60.000 (3)
C11B—C10—S3B	111.7 (9)	O3—C18B—H18B	100.8
C1—C10—S3B	120.5 (3)	C19—C18B—H18B	90.9
C11A—C10—S3A	108.9 (6)	C19^iii^—C18B—H18B	141.7
C11B—C10—S3A	10.3 (9)	C18B^iv^—C18B—H18B	87.3
C1—C10—S3A	122.2 (3)	C18B^iii^—C18B—H18B	144.3
S3B—C10—S3A	116.0 (3)	O3—C18B—H18C	108.8
C10—C11A—C12	111.6 (9)	C19—C18B—H18C	110.2
C10—C11A—H11A	124.2	C19^iii^—C18B—H18C	152.2
C12—C11A—H11A	124.2	C18B^iv^—C18B—H18C	109.4
C13—C12—C11A	108.4 (6)	C18B^iii^—C18B—H18C	155.6
C13—C12—S3B	118.9 (4)	H18B—C18B—H18C	24.0
C11A—C12—S3B	12.2 (5)	O3—C18B—H18D	112.3
C13—C12—H12	125.8	C19—C18B—H18D	112.8
C11A—C12—H12	125.8	C19^iii^—C18B—H18D	68.2
S3B—C12—H12	115.0	C18B^iv^—C18B—H18D	140.5
C12—C13—C11B	109.3 (9)	C18B^iii^—C18B—H18D	81.4
C12—C13—S3A	117.1 (4)	H18B—C18B—H18D	132.3
C11B—C13—S3A	12.1 (8)	H18C—C18B—H18D	109.6
C12—C13—H13	121.4	C19^iii^—C19—C19^iv^	59.998 (1)
C11B—C13—H13	128.3	C19^iii^—C19—C18A	104.8 (8)
S3A—C13—H13	121.4	C19^iv^—C19—C18A	102.0 (8)
C13—S3A—C10	93.6 (3)	C19^iii^—C19—C18B	68.6 (14)
C10—C11B—C13	106.0 (16)	C19^iv^—C19—C18B	100.6 (9)
C10—C11B—H11B	127.0	C18A—C19—C18B	41.9 (12)
C13—C11B—H11B	127.0	C19^iii^—C19—C18B^iv^	94.9 (9)
C12—S3B—C10	93.3 (3)	C19^iv^—C19—C18B^iv^	59.6 (11)
C17—O2—C14	107.3 (3)	C18A—C19—C18B^iv^	45.4 (9)
C17—O2—Tb	128.35 (19)	C18B—C19—C18B^iv^	70.3 (9)
C14—O2—Tb	124.1 (2)	C19^iii^—C19—H19A	110.7
O2—C14—C15	106.8 (3)	C19^iv^—C19—H19A	56.0
O2—C14—H14A	110.4	C18A—C19—H19A	111.3
C15—C14—H14A	110.4	C18B—C19—H19A	144.3
O2—C14—H14B	110.4	C18B^iv^—C19—H19A	74.3
C15—C14—H14B	110.4	C19^iii^—C19—H19B	111.0
H14A—C14—H14B	108.6	C19^iv^—C19—H19B	147.9
C14—C15—C16B	107.1 (5)	C18A—C19—H19B	110.1
C14—C15—C16A	94.9 (5)	C18B—C19—H19B	103.9
C16B—C15—C16A	25.4 (6)	C18B^iv^—C19—H19B	149.6
C14—C15—H15A	112.8	H19A—C19—H19B	108.9
C16B—C15—H15A	124.1	C19^iii^—C19—H19C	171.1
C16A—C15—H15A	112.8	C19^iv^—C19—H19C	111.4
C14—C15—H15B	112.8	C18A—C19—H19C	74.0
C16B—C15—H15B	87.4	C18B—C19—H19C	113.0
C16A—C15—H15B	112.8	C18B^iv^—C19—H19C	77.9
H15A—C15—H15B	110.2	H19A—C19—H19C	62.6
C15—C16A—C17	101.5 (7)	H19B—C19—H19C	77.4
C15—C16A—H16A	111.5	C19^iii^—C19—H19D	77.4
C17—C16A—H16A	111.5	C19^iv^—C19—H19D	111.4
C15—C16A—H16B	111.5	C18A—C19—H19D	141.5
C17—C16A—H16B	111.5	C18B—C19—H19D	111.1
H16A—C16A—H16B	109.3	C18B^iv^—C19—H19D	170.6
C15—C16B—C17	103.5 (6)	H19A—C19—H19D	103.1
C15—C16B—H16C	111.1	H19B—C19—H19D	39.8
C17—C16B—H16C	111.1	H19C—C19—H19D	109.3
C15—C16B—H16D	111.1		

Symmetry codes: (i) −*y*+1, *x*−*y*, *z*; (ii) −*x*+*y*+1, −*x*+1, *z*; (iii) −*y*+2, *x*−*y*, *z*; (iv) −*x*+*y*+2, −*x*+2, *z*.
